# *KIT* Mutant/Core binding factor-negative acute myeloid leukemia might be a complex subgroup with dismal prognosis: a single-center retrospective analysis

**DOI:** 10.1007/s00277-026-06814-7

**Published:** 2026-01-20

**Authors:** Rui Jiang, Zhibo Zhang, Yizi Liu, Wenqiang Qu, Zhao Zeng, Linlin Wang, Qian Wang, Jia Yin, Suning Chen

**Affiliations:** 1 National Clinical Research Center for Hematologic Diseases, Jiangsu Institute of Hematology, First Affiliated Hospital of Soochow University, Suzhou, China; 2 Institute of Blood and Marrow Transplantation, Collaborative Innovation Center of Hematology, Soochow University, Suzhou, China; 3 Department of Hematology, The First People’s Hospital of Yancheng, Affiliated Hospital of Nanjing University Medical School, The Yancheng Clinical College of Xuzhou Medical University, Yancheng, China

**Keywords:** KIT, AML, Core binding factor, CEBPA, Venetoclax

## Abstract

*KIT* mutations are well-established as poor prognostic markers in core binding factor AML (CBF AML). However, data on *KIT* mutation in CBF-negative (CBF-neg) AML remains scarce. This retrospective study aimed to characterize the clinical features and outcomes of patients with *KIT* mutant (*KIT* mut)/CBF-neg AML. We conducted a retrospective study in our single center and identified non-M3 de novo AML with KIT mutations by next-generation sequencing (NGS) from January 2018 to June 2024. Core binding factor (CBF) AML or patients with underlying systemic mastocytosis were excluded. The clinical data of *KIT* mut/CBF-neg AML was collected and analyzed. 45 patients were enrolled in the cohort, including 3 patients with secondary AML. The median variant allele frequency (VAF) of *KIT* mutation was 41.2% and the most frequent comuations were *CEBPA* (27/45, 60%), *WT1* (12/45, 26.7%) and *NRAS* (11/45, 24.4%). After a median follow-up of 48 months, the median event-free survival (EFS) and overall survival (OS) of the cohort was 15.3 months and 24.1 months, respectively. Patients with *KIT* exon 17 mutations tended to harbor an inferior EFS and OS. In conclusion, *KIT* mutant/CBF-neg AML was a complex subgroup with dismal prognosis and NIT might bring benefits for those patients.

**Supplementary Information:**

The online version contains supplementary material available at 10.1007/s00277-026-06814-7.

## Key points

KIT mutant/CBF-neg AML was a complex subgroup with various molecular biological features and CEBPA as most frequent comutation.

KIT mutant/CBF-neg AML harbored a dismal survival which was improved by allo-HSCT.

**Supplementary Information:**

The online version contains supplementary material available at 10.1007/s00277-026-06814-7.

## Introduction

KIT, also known as stem cell factor (SCF) receptor, is one of prominent member in the family of Type III tyrosine kinase receptor [1]. The *KIT* gene is a proto-oncogene located at the 11 locus of long arm of chromosome 4 [2], and the gene product physiologically is involved in cellular proliferation and survival, cell differentiation, chemotaxis and activation of gene transcription, by participating in several signaling pathways, including PI3K pathway, JAK/STAT pathway, Src pathway and MAPK pathway [1–6].

*KIT* dysfunction, induced by gain-of function mutations, plays an important role in tumorigenesis and progression. It has been reported *KIT* mutation occurred in several solid cancers, including thymic cancer, breast cancer and gastrointestinal stromal tumor (GIST) [1, 7]. In myeloid neoplasms (MNs), *KIT* mutation is primarily correlated with systemic mastocytosis (SM), with 90% patients harboring activating mutation and *KIT* D816V occurred most frequently [8]. Aside from SM, acute myeloid leukemia (AML) was the most common MNs with *KIT* mutation, which was detected in 4%−6% de novo AML [9, 10], most frequently occurred in core binding factor AML (CBF AML).

Previous study has primarily focused on the prognosis of *KIT* mutation in CBF AML. It has been well established that *KIT* mutation was one of poor prognostic factors in CBF AML treated by intensive chemotherapy. Moreover, *KIT* exon 17 mutations were associated with unsatisfactory survival outcome in patients with *RUNX1::RUNXT1* AML receiving hypomethylating agents (HMA) [11]. However, less attention has been given to CBF-negative (CBF-neg) AML, resulting in a lack of understanding regarding the distribution and prognosis of *KIT* mutation in the specific population. Consequently, we performed a retrospective study on patients with *KIT* mutant (*KIT* mut)/CBF-neg AML and investigated the clinical characteristics, treatment procedures and survival to provide preliminary clues into the individual treatment strategy of the subgroup.

## Materials and methods

### Patients

A total of 45 newly-diagnosed, non-M3 AML patients who presented *KIT* mutation via next-generation sequencing (NGS), without fusion gene *RUNX1::RUNXT1* or *CBFB::MYH11* detected by conventional cytogenetic analysis or RT-PCR between January 2018 and June 2024 in our single center were retrospectively analyzed. As *KIT* activating mutation or *KIT* D816V is one of diagnostic criteria in SM based on the 5th edition of the World Health Organization classification of MNs [12], patients with evidence of underlying SM were excluded (see Supplemental Fig. 1). This study was approved by the responsible ethics committees and performed in accordance with the Declaration of Helsinki. Informed consent was obtained from all patients involved in the study.

### Treatment procedures and measurable residual disease (MRD) assessment

Forty-three patients received at least one cycle of induction therapy and were included for response assessment. Induction therapy was classified into two groups: non-intensive therapy (NIT) and intensive therapy (IST). NIT included venetoclax + HMA (decitabine or azacytidine) ± others (homoharringtonine or all-trans-retinoic acid). IST included standard regimen [IA (idarubicin, cytarabine) and DA (daunorubicin, cytarabine) ± venetoclax and priming regimen, including IAG [idarubicin, doxorubicin, cytarabine, human granulocyte colony stimulating factor (G-CSF)], HAAG (homoharringtonine, doxorubicin, cytarabine, G-CSF) and ECAG (aclarubicin, doxorubicin, cytarabine and G-CSF). Response assessment was conducted according to the 2022 recommendation of the European LeukemiaNet (ELN 2022) [13]. MRD assessment was performed by multiparameter flowcytometry or quantitative PCR (qPCR). MRD negativity was defined as < 0.1% CD45-expressing cells. NPM1 > 0.2% variant allele frequency (VAF) detected by qPCR was considered MRD positive [14]. Twenty patients underwent allogeneic hematopoietic stem cell transplantation (allo-HSCT).

### Survival definition and statistical analysis

Event-free survival (EFS) was calculated from the date of diagnosis as AML to the date of treatment failure, relapse or death induced by any cause, whichever happened first, and relapse-free survival (RFS) was calculated from the date of remission to the date of relapse or death, whichever happened first. Overall survival (OS) was defined as the duration between the date of diagnosis and the date of death.

Categorical variables were summarized using numbers (proportion) and compared with Chi-square test and Fisher’s exact test. Continuous variables were summarized using median (range) and compared with Mann-Whitney test. Survival curves were constructed with Kaplan-Meier method and survival analysis between different subgroups was conducted by Log-rank test. Predictors for EFS and OS was performed by univariate Cox hazard analysis and multivariable analysis selected variables with *p* value of < 0.1 in univariate analysis and clinical significance. The proportional hazards assumption was assessed using Schoenfeld residuals and log-minus-log plots, and no significant violation was observed. *P* value of < 0.05 was considered statistically significant. IBM SPSS 26.0 and Graphpad Prism 9 were used for statistical analysis.

## Results

### Incidence, type and location of *KIT* mutation in *KIT* mutant/CBF-neg AML

**Fig. 1:**
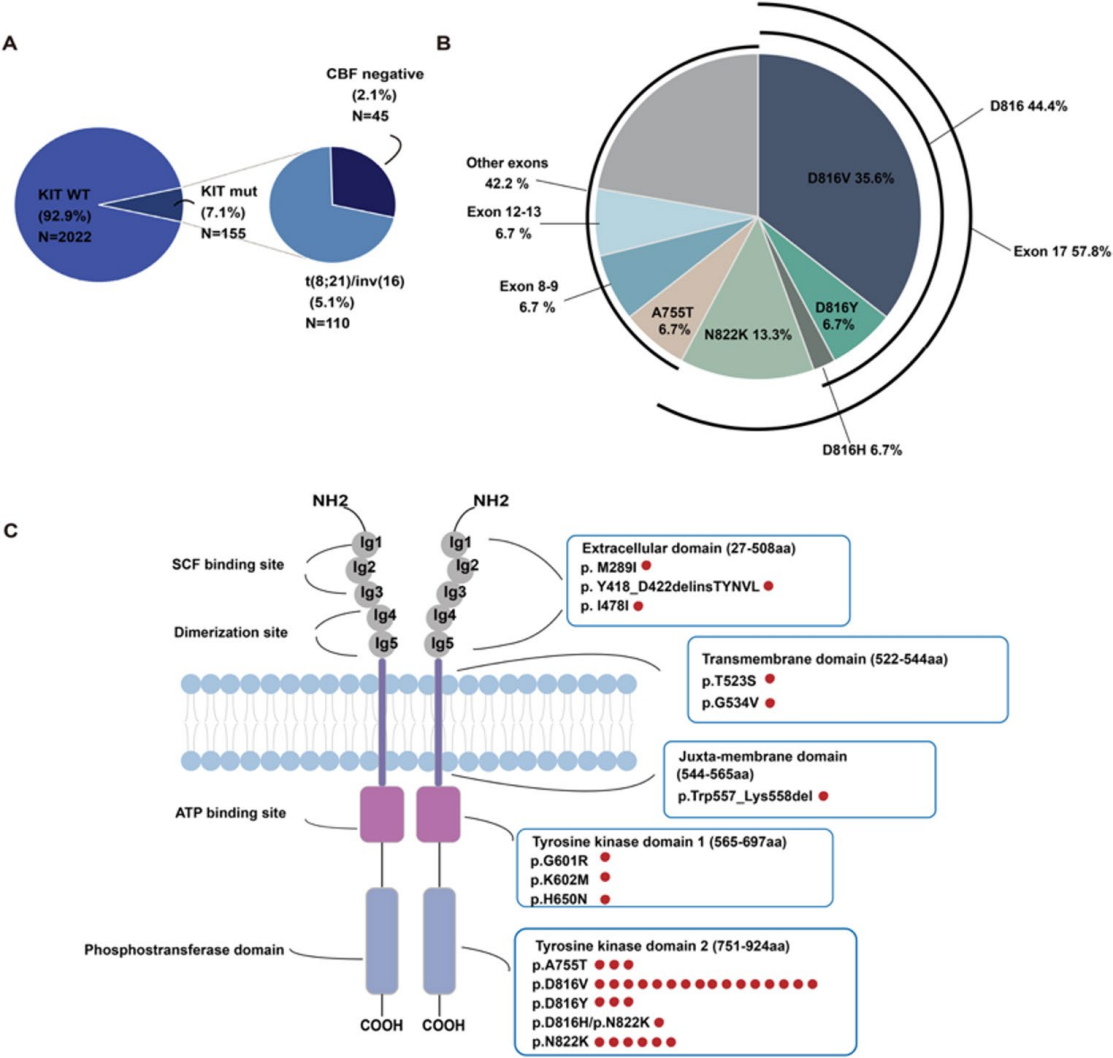
Characteristics of *KIT* mutation in 45 *KIT* mut/CBF-neg AML (**A**) Distribution and frequency of *KIT* mutation in de-novo AML. (**B**) Pie chart showing type and frequency of *KIT* mutation in the 45 patients. (**C**) Schematic representation of *KIT* domains depicting type and number of mutations of the cohort

From January 2018 to June 2024, we reviewed 2, 177 patients diagnosed as AML and detected 155 patients (7.1%) with *KIT* mutation by NGS, including 110 (5.1%) patients with fusion gene *RUNX1::RUNXT1* or *CBFB::MYH11* and 45 patients (2.1%) without such fusion gene (Fig. 1A). By further analysis of the 45 KIT mut/CBF-neg patients, the most common mutation was at exon 17 (26/45, 57.8%). *KIT* D816V was most frequently seen with an incidence of 35.6% (16/45), followed by *KIT* N822K with a percentage of 13.3% (7/45) (Fig. 1B). Furthermore, we noted D816H was in coexistence with N822K in one patient (Fig. 1C).

### Clinical characteristics

The median age at diagnosis of 45 patients with *KIT* mut/CBF-neg was 46 years old (range: 17–74 years old), including 3 patients with secondary AML, among which one patient was diagnosed as therapy-related AML and two patients progressed from MDS and MPN, respectively. Twenty-six patients (26/45, 57.8%) exhibited normal karyotype, six patients (6/45, 13.3%) exhibited myelodysplasia-related cytogenetic abnormalities, and 2 patients (2/45, 4.4%) presented *KMT2A* rearrangement. Fourty-three patients were available for analysis of ELN 2022 risk stratification at diagnosis: 46.5%, 30.2% and 23.3% in favorable, intermediate, and adverse, respectively. The detailed clinical characteristics of the cohort was listed in Table 1.

**Table 1:** Clinical characteristics of 45 *KIT* mutant/core binding factor (CBF)-negative (CBF neg) AML patients

Variables	All patients (*n* = 45)
Sex, *n* (%)	
Male	30 (66.7)
Female	15 (33.3)
AML type, *n* (%)	
De novo	42 (93.3)
Secondary	3 (6.7)
Age (years), median (range)	46 (17–74)
CBC at diagnosis, median (range)	
WBC, ×10^9^/L	41.7 (2.8–239.5.8.5)
Hb, g/L	89 (48–134)
PLT, ×10^9^/L	30 (3–1466)
BM blast (%), median (range)	65 (13–92)
*KMT2A* rearrangement, *n* (%)	2 (4.4%)
AML with MRC, *n* (%)	13 (28.9%)
ELN 2022 risk stratification (*n* = 43), *n* (%)	
Favorable	20 (46.5)
Intermediate/Adverse	23 (53.5)
*KIT* mutational site, *n* (%)	
*KIT*−816	20 (44.4)
*KIT*-D816V	16 (35.6)
*KIT*-N822K	7 (15.6)
Comutations, *n* (%)	
*CEBPA*	27 (60)
*NPM1*	6 (13.3)
*FLT3*-ITD	3 (6.7)
*FLT3*-TKD	3 (6.7)
*IDH*1/2	6 (13.3)
*TP53*	2 (4.4)

*ANC* absolute neutrophil count, *BM *bone marrow, *CBC *complete blood count, *Hb *hemoglobin, *MRC *myelodysplasia-related changes, *PLT * platelet, *WBC* white blood cell count

### Comutations

**Fig. 2:**
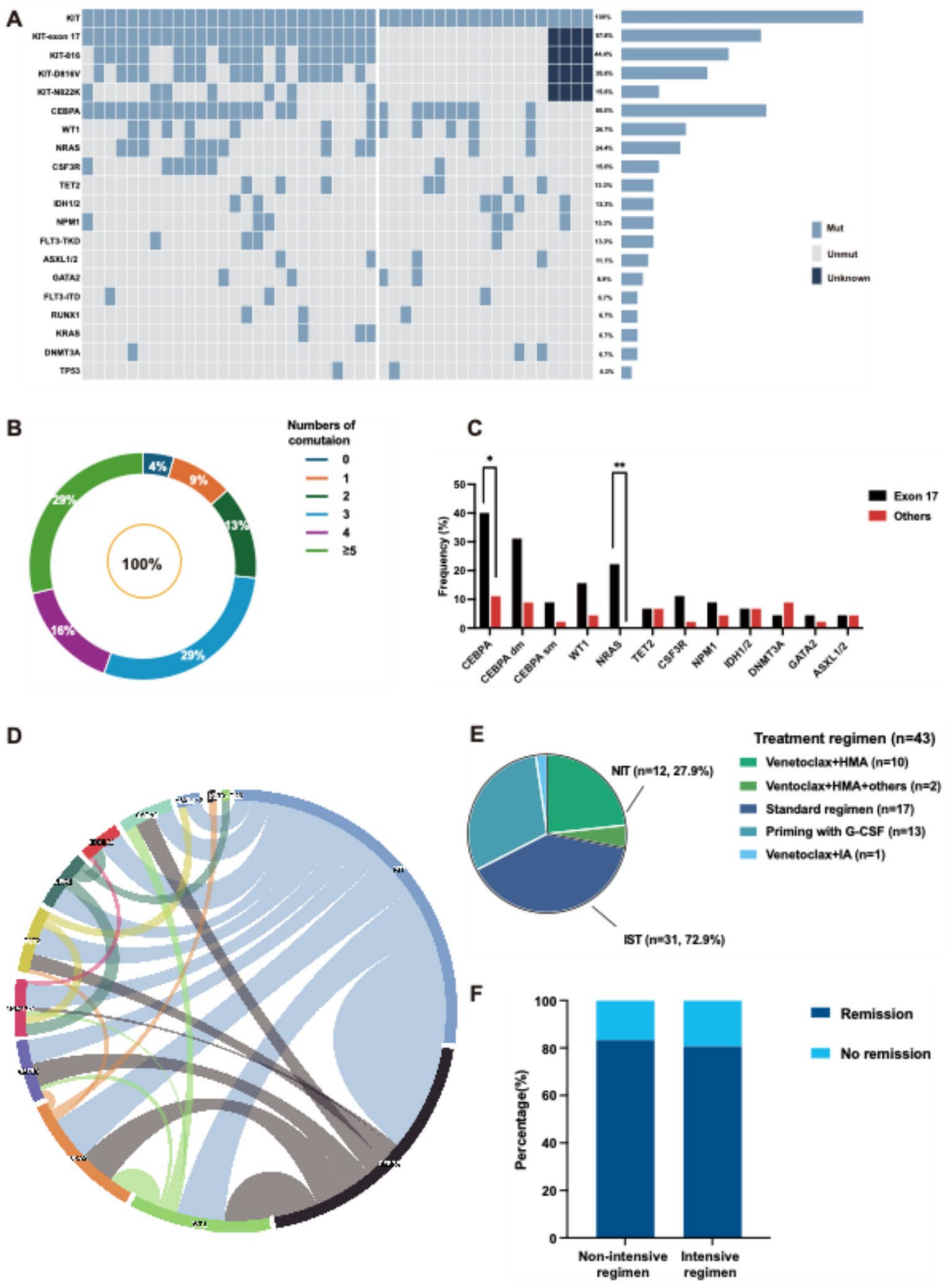
Co-mutation analysis, treatment disposition and response assessment of the cohort (**A**) Mutational landscape of 45 *KIT* mut/CBF-neg AML. Each column represents one patient, and each line represents one gene. (**B**) Proportions of the number of comutations of the cohort. (**C**) Comparison of the frequencies of comutations between patients with *KIT* exon 17 mutation (*n* = 26) and with others (*n* = 15). Comparisons were made by Chi-square test and Fisher’s exact test and considered statistically significant at *p* values less than 0.05. (**D**) Circos program of the co-occurrence and frequency of mutational genes. (**E**) Distributions of induction therapy with various treatment procedures. (**F**) Bar chart showing the rate of remission of induced with non-intensive therapy (NIT) (*n* = 12) and intensive therapy (IST) (*n* = 31). * *p* < 0.05; ** *p* < 0.001

The mutational profile of 45 patients with *KIT* mut/CBF-neg was depicted in Fig. 2A. The median variant allele frequency (VAF) of *KIT* mutation was 41.2% (range: 1%−64.5%) and the median number of comutations except *KIT* mutation was three (range: 0–7). The most common mutated genes were *CEBPA* (27/45, 60%), *WT1* (12/45, 26.7%), *NRAS* (11/45, 24.4%), *TET2* (7/45, 15.6%), *CSF3R* (6/45, 13.3%), *NPM1* (6/45, 13.3%), *IDH1/2* (6/45,13.3%), *DNMT3A* (6/45, 13.3%) and *GATA2* (5/45, 11.1%) (Fig. 2A). Compared with CBF AML, *CEBPA*, *WT1*, *NPM1* and *IDH1/2* cooccurred more frequently in *KIT* mut/CBF-neg AML with statistically significance (see Supplemental Fig. 2**)**. Furthermore, among patients with CEBPA comutations, 74.1% (20/27) harbored CEBPA^dm^. Twenty-five patients were available for further analysis of mutational sites, including seventeen patients (44.4%, 17/25) with bZIP in-frame (bZIP-inf) mutations (see Supplemental Fig. 2). Only 4.4% (2/45) patients showed single *KIT* mutation, including one patient solely with *KIT* D816V and the other with deletion/insertion of extracellular domain. Most patients (39/45, 86.7%) exhibited more than two comutations except *KIT*, among which 28.8% (13/45) possessed more than 5 comutations (Fig. 2B).

### Comparison of baseline clinical features between patients with *KIT* exon-17 mutation and with others

Considering exon 17 was the major mutant site in AML as reported before [15, 16], we further divided 41 patients, available for analysis of *KIT* mutation site, into two groups: patients with exon 17 (*n* = 25) and others (*n* = 16) and made comparison between the subgroups (Table 2). The proportion of male was significantly higher in patients with exon 17 (76.9% vs. 40.0%, *P =* 0.04). Meanwhile, patients with *KIT* exon 17 showed a significantly lower median VAF (29.1% vs. 48.3%, *P =* 0.0005). Additionally, the frequency of several somatic mutations was relatively higher in the subgroup of patients with exon 17, including *CEBPA*, *WT1*, *NRAS*, *CSF3R* and *NPM1* (Fig. 2C and D), among which the incidence of *CEBPA* and *NRAS* was significantly higher in patients with exon 17 (CEBPA: 40.0% vs. 11.1%, *p =* 0.025; NRAS: 22.2% vs. 0.0%, *p =* 0.006) (Fig. 2C).

**Table 2:** Clinical characteristics between patients with KIT-exon 17 and others

Variables	Exon 17 (*n* = 26)	Others (*n* = 15)	*p*
Male, *n* (%)	20 (76.9)	6 (40.0)	**0.04**
Age (years), median (range)	57 (17–74)	47 (18–65)	0.38
CBC at diagnosis, median (range)			
WBC, ×10^9^/L	51.5 (3.2–239.5.2.5)	6.7 (0.3–48.7)	**< 0.001**
Hb, g/L	92 (34–129)	83 (57–119)	0.72
PLT, ×10^9^/L	29 (8–1466)	52 (8–175)	0.19
BM blast (%), median (range)	65 (15–88)	51 (13–92)	0.49
*KIT* VAF (%), median (range)	29.1 (2.1–64.5)	48.3 (4.1–63.8)	**0.0005**
ELN 2022 risk stratification, *n* (%)			0.30
Favorable	8 (30.8)	3 (20.0)	
Intermediate	10 (38.5)	6 (40.0)	
Adverse	4 (15.4)	6 (40.0)	
Gene mutation, *n* (%)			
*CEBPA*	18 (69.2)	5 (33.3)	**0.02**
*NRAS*	10 (38.5)	0 (0.0)	**0.0067**
*NPM1*	4 (15.4)	2 (13.33)	> 0.99
Induction regimen, *n* (%)			**0.03**
NIT	4 (15.4)	7 (46.7)	
IST	21 (80.8)	7 (46.7)	
Allo-HSCT, *n* (%)	9 (34.6)	8 (50)	0.35

*Allo-HSCT *allogeneic hematopoietic stem cell transplantation, *IST *intensive therapy, *NIT *non-intensive therapy (NIT), *VAF *variant allele frequency

### Treatment disposition and response rate

Two patients refused to receive induction therapy and were excluded for response assessment. Forty-three patients received at least one cycle of induction therapy and available for response assessment: 27.9% (12/43) patients received NIT, including ten patients receiving venetoclax and HMA, and the others (31/43, 72.1%) received IST. The proportion of various induction regimen was depicted in Fig. 2E. Comparisons of baseline clinical features, response and MRD assessment between patients with different treatment disposition were listed in Supplemental Table 1. The rate of complete remission (CR) after induction therapy was comparable between patients with NIT and with IST (83.3%, 10/12 vs. 80.6%, 25/31) (Fig. 2F). Thirty-four patients were available for MRD analysis after one cycle of induction therapy. 47.1% (16/34) patients achieved MRD negativity, including ten patients with NIT after one cycle. Twenty patients (44.4%) patients underwent subsequent allo-HSCT, including 5 patients who failed to maintain remission and collapsed after a median time of 10.6 months (range: 5.1–18 months) since allo-HSCT.

### Survival and prognostic analysis

**Fig. 3:**
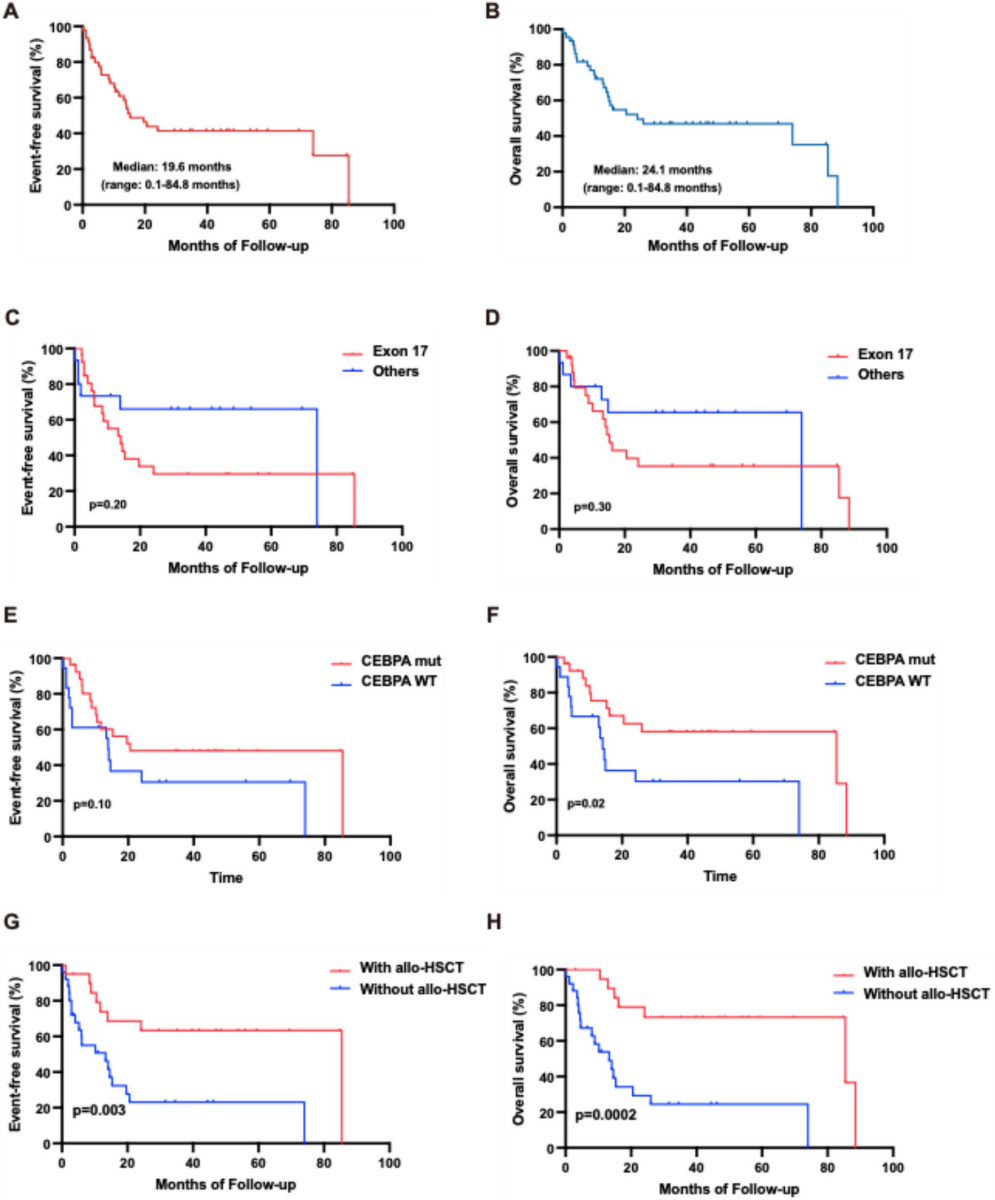
Survival analysis of the cohort (**A**-**B**) Kaplan-Meier curves of event-free survival (EFS) and overall survival (OS) of 45 *KIT* mut/CBF-neg AML. (**C**-**D**) Kaplan-Meier curves of EFS and OS of patients with *KIT* exon 17 mutation and with others. (**E**-**F**) Kaplan-Meier curves of EFS and OS of patients with *CEBPA* mutation and WT. (**G**-**H**) Kaplan-Meier curves of EFS and OS of patients with allogeneic hematopoietic stem cell transplantation (allo-HSCT) and without

The last follow-up was completed by December 2025. With a median follow-up of 48 months, the median EFS and OS of the cohort were 15.3 months (range: 0.1–85.4 months) (Fig. 3A) and 24.1 months (range: 0.1–88.5 months) (Fig. 3B). Patients with *KIT* mutation at exon 17 showed an inferior EFS (median: 14.1 vs. 74 months) (Fig. 3C) and OS (median: 15.3 vs. 74 months) (Fig. 3D) in comparison with other sites. Moreover, patients with *KIT* exon-17 mutation harbored a significantly poor RFS (median: 12.2 vs. 72.2 months; *P* = 0.03) (see supplemental Fig. 4). Meanwhile, patients with *CEBPA* mutation appeared to have an advantage of EFS (median: 20.6 vs. 13.9 months; *P =* 0.10) over those with wildtype [17] (Fig. 3E), while a significantly superior OS was seen in the subgroup of patients with *CEBPA* comutation (median: 85.4 vs. 14.1 months; *P =* 0.02) (Fig. 3F). Furthermore, patients with *CEBPA* bZIP in-frame mutations (bZIP-inf) showed superiority of EFS and OS over patients with *CEBPA* WT [17]. However, no obvious difference of survival was seen between patients with *CEBPA* bZIP-inf and with other mutational sites (see supplemental Fig. 5). There was no significant difference of OS and EFS between patients with *NPM1* mutation and *NPM1* WT despite a superior EFS in patients with *NPM1* mutation (see supplemental Fig. 5). According to ELN2022 risk stratification, patients with favorable risk harbored an advantage of EFS and OS over patients with intermediate or adverse (median EFS: 85.4 months vs. 13.9 months, *P =* 0.11; median OS: 85.4 vs. 14.6 months, *P =* 0.03) (see supplemental Fig. 5). Allo-HSCT significantly improved EFS (median: 85.4 vs. 13.9 months; *P =* 0.003) (Fig. 3G) and OS (median: 85.4 vs. 14.6; *p =* 0.0002) (Fig. 3H) in the cohort.

We further performed univariate and multivariate cox regression analysis to better clarify the cohort (Tables 3 and 4), and identified *TP53* mutation at diagnosis was an independent risk factor (EFS: hazard ratio [HR], 11.15 [95% CI: 2.12–58.72], *P =* 0.004; OS: HR, 8.75 [95% CI: 1.41–54.45], *P =* 0.02) while allo-HSCT was an independent protective factor (EFS: HR, 0.34 [95% CI: 0.13–0.87], *P =* 0.025; OS: HR, 0.18 [95% CI: 0.05–0.71], *P =* 0.014). Moreover, *NPM1* mutation was an independent risk factor for EFS (HR: 4.19 [95% CI: 1.47–11.96], *P =* 0.007) while intermediate or adverse risk stratified by ELN 2022 was an an independent risk factor for OS (HR: 4.23 [95% CI: 1.05–17.17], *P =* 0.043). We identified *CEBPA* mutation as a significant protective factor (HR: 0.39 [95% CI: 0.17–0.89], *P =* 0.026) and *NPM1* mutation as a significant risk factor (HR: 3.30 [95% CI: 1.08 ~ 10.06], *P =* 0.04) for OS in univariate analysis, but not significant in multivariate analysis.

**Table 3:** Univariate and multivariate Cox regression analysis of event-free survival (EFS)

Variables	Univariate		Multivariate	
	Hazard ratio (95% CI)	*p*	Hazard ratio (95% CI)	*p*
Male	1.34 (0.56–3.22)	0.51		
Age	1.01 (0.98–1.03)	0.60		
WBC	1.00 (0.99–1.00.99.00)	0.26		
Secondary AML	1.05 (0.25–4.45)	0.95		
Intermediate/adverse risk^*^	1.92 (0.84–4.35)	0.12		
*KIT* exon 17 mut	1.83 (0.72–4.68)	0.21		
*CEBPA* mut	0.53 (0.25–1.20)	0.13		
*CEBPA* bZIP-inf mut	0.84 (0.37–1.91)	0.68		
*NPM1*mut	**4.1 (1.47–11.42)**	**0.007**	**4.19 (1.47–11.96)**	**0.007**
*TP53* mut	**12.27 (2.46–61.30)**	**0.002**	**11.15 (2.12–58.72)**	**0.004**
Allo-HSCT	**0.29 (0.12–0.70)**	**0.006**	**0.34 (0.13–0.87)**	**0.025**

bZIP-inf: bZIP in-frame mutations; ^*^: stratified by ELN 2022

**Table 4:** Univariate and multivariate Cox regression analysis of overall survival (OS)

Variables	Univariate		Multivariate		
	Hazard ratio (95% CI)	*p*		Hazard ratio (95% CI)	*p*
Male	1.05 (0.43–2.57)	0.92			
Age	1.01 (0.98–1.04)	0.40			
WBC	1.00 (0.99–1.00.99.00)	0.65			
Secondary AML	1.32 (0.31–5.65)	0.71			
Intermediate/adverse risk^*^	**2.50 (1.02–6.09)**	**0.04**		**4.24 (1.05–17.17)**	**0.04**
*KIT* exon 17 mut	1.65 (0.64–4.27)	0.31			
*CEBPA* mut	**0.39 (0.17–0.89)**	**0.03**		1.43 (0.42–4.89)	0.57
*CEBPA* bZIP-inf mut	0.65 (0.27–1.59)	0.35			
*NPM1*mut	**3.30 (1.08 ~ 10.06)**	**0.04**		2.05 (0.54 ~ 7.73)	0.29
*TP53* mut	**23.77 (3.91–144.42.91.42)**	**< 0.001**		**8.75 (1.41–54.45)**	**0.02**
Allo-HSCT	**0.18 (0.07–0.50)**	**0.001**		**0.18 (0.05–0.71)**	**0.014**

^*^: stratified by ELN 2022

## Discussion

Here we reported the distribution of *KIT* mutation in CBF-neg AML and clarified the clinical characteristics and prognosis of the subgroup. Our results indicated that *KIT* mut/CBF-neg AML might possess a dismal survival, regardless comutation and allo-HSCT might benefit the underestimated populations.

It has been an important issue concerning the prognosis of *KIT* mutation in AML, especially in CBF-AML, including AML with *RUNX1::RUNXT1* and *CBFB::MYH11*. The incidence of *KIT* mutation was approximately 4%−6% in AML [9, 10], which frequently occurred in 10%−40% CBF-AML, with a poor survival in the subgroup of patients with *RUNX1::RUNXT1* based on multiple retrospective and perspective clinical studies [11, 15, 18–22]. However, there was few research on *KIT* mutation in CBF-neg AML. A retrospective study preliminarily presented the distribution of *KIT* mutation in myeloid neoplasms, including 56% patients with CBF-neg AML and 45% with CBF-AML [16]. A real-word study showed that in comparison with CBF-AML, CBF-neg AML showed a superior survival with a 3-year OS of 77.3% and a 3-year DFS of 73.6%. However, the study failed to focus on the subgroup of *KIT* mut/CBF-neg AML, meanwhile, *KIT* mutation showed no prognostic significance in the study [23]. Whereas based on two AML independent databases, a retrospective study identified 17 *KIT* mut/CBF-neg AML with a poor median OS of 26.4 months. Recently, another study identified 16 patients of the subgroup and pointed out the subgroup showed extremely poor survival with a median OS of 9 months and a median RFS of 3.2 months, regardless comutations [24]. Consistent with the previous studies, our study indicated that *KIT* mut/CBF-neg AML was a specific subgroup and showed a poor prognosis with a median EFS of 15.3 months and a median OS of 24.1 months, despite most patients categorized into favorable-risk and intermediate-risk subgroup based on ELN2022.

In this study, we first depicted the clinical features and the mutational landscape of *KIT* mut/CBF-neg patients in AML. Three secondary AML was included and by further analysis, secondary AML was not significantly associated with EFS and OS in our univariate analysis. Several studies reported that the incidence of *CEBPA* mutation was relatively low in adult patients with CBF AML, approximately 1% [21, 23]. Unlike that in CBF AML, *CEBPA* mutation seemed to be more frequent in CBF-neg AML, with an incidence of 36.4% according to a retrospective study [23]. In line with that, our study discovered that over 50% of the subgroup presented *CEBPA* mutation. By further analysis, it was unexpected that patients with *CEBPA* appeared to attain a superior survival in the subgroup and *CEBPA* mutation had a significantly positive impact on OS, which was not mentioned in the previous study. Moreover, the incidence of *NPM1* mutation was relatively lower in our cohort than that reported before, which was detected in 38% patients [24]. In our cohort, *NPM1* showed a significantly negative impact on EFS and OS in univariate cox regression, which was inconsistent with Log-rank test, a possible explanation maybe a small sample size of *NPM1* mutation in the rare group. However, the rarity of this co-mutation pattern means its implications remain to be fully elucidated. We noticed TP53 mutational status at diagnosis was negatively associated with EFS and OS although two patients in our cohort harbored TP53 mutation, one of which underwent a course of venetoclax plus decitabine as induction, achieved CR with undetectable MRD, received allo-HSCT after consolidation with intermediate dose cytarabine and maintained CR after a follow-up of 40.8 months while the other one received a course of venetoclax plus decitabine as induction, achieved CR but relapsed after 4.8 months and collapsed.

Aside from description of clinical characteristics, we preliminarily probed into the efficiency of induction therapy in the rare subgroup. We noticed a comparable remission rate between patients with NIT and IST however a relatively higher rate of MRD elimination in patients with NIT. Notably, one patient who received venetoclax in combination with IA as induction therapy remained remission after a follow-up of 34.5 months. A systemic review reported that there was a statistically significant superiority of survival in those who achieved MRD negativity. Moreover, in those who achieved morphological remission, MRD negativity was positively associated with disease-free survival (DFS) and OS [25]. Accordingly, our retrospective study provided preliminary evidence that venetoclax-based regimen might be an appropriate choice for the rare subgroup due to the achievement of a fast MRD elimination, which might make for future survival benefit.

Nonetheless, there might be some limitations in our study. First, the sample size of our cohort was small which might result in the instability of cox regression analysis and overestimated the influence of variables, for example *NPM1* and *TP53*, a rare comutation pattern needed further study. Second, due to retrospective study, the choice of induction regimen and whether to perform allo-HSCT were dependent on physician and the economic condition of the patient. Moreover, a small sample size of NIT subgroup, we failed to perform propensity score–matched analysis and our findings should be considered exploratory. We performed the comparison of clinical features between patients with NIT and IST, and there was no evident imbalance between the two subgroups, except *NPM1* mutation and *KIT* mutational site. It has been elucidated that *NPM1* mutation was associated with favorable response rate and survival while *KIT* mutation was associated poorer survival in several real-word study of venetoclax plus HMA [26, 27]. Accordingly, a higher MRD elimination rate in NIT might be attributable to the higher incidence of *NPM1* mutation. Due to the limited sample size in NIT subgroup, we might overstate the clinical effect and survival benefit of NIT, which might be ascribed to the imbalance in the rate of patients with *KIT* exon 17 mutation between NIT and IST and might partly explain the contradiction on the role of venetoclax-based regimen in KIT mut AML between our study and the previous study [28]. Moreover, our study failed to answer whether venetoclax-based regimen could induce a sustainable survival benefit in *KIT* mut/CBF-neg AML, which needs prospective study with large sample size and long-term follow-up.

In conclusion, our results indicated that *KIT* mut/CBF-neg AML was a complex subgroup, which might be overlooked in our clinical practice, presented various molecular biological features and harbored a dismal prognosis. Our findings raised a presumption that induction with venetoclax + HMA might be appropriate for those patients, which might induce an early MRD elimination and remission, subsequently bridging to allo-HSCT and attain a sustainable remission. Our research is limited due to small sample size base on a retrospective study from our single center. There is a great need in future to investigate the characteristics and treatment strategies of *KIT* mut/CBF-neg AML and develop new markers for monitoring MRD by conducting a large prospective study.

## Supplementary Information

Below is the link to the electronic supplementary material.

Supplementary file 1 (DOCX 10.5 MB)

Supplementary file 2 (DOCX 28.0 KB)

## Data Availability

The data involved in this study are available from the corresponding authors, Jia Yin (yinjia@suda.edu.cn) and Suning Chen (chensuning@suda.edu.cn) upon reasonable request.
