# Supplementary material for: KIT Mutant/Core binding factor-negative acute myeloid leukemia might be a complex subgroup with dismal prognosis: a single-center retrospective analysis
Source: Ann Hematol. 2026 Jan 20;105(2):42. doi: 10.1007/s00277-026-06814-7 (PMC12819538; doi:10.1007/s00277-026-06814-7)
Supplement: Supplementary file 1 — (DOCX 10.5 MB) [file 277_2026_6814_MOESM1_ESM.docx]

**Title:** *KIT* Mutant/Core Binding Factor-Negative Acute Myeloid Leukemia might be a distinct subgroup with dismal prognosis: a single-center retrospective analysis

**Journal:** Annals of Hematology

**Authors:**

Rui Jiang^1, 2#^, Zhibo Zhang^1, 2#^, Yizi Liu^1, 2#^, Wenqiang Qu^1, 2^, Zhao Zeng^1, 2^ , Linlin Wang^3^, Qian Wang^1, 2^, Jia Yin^1, 2^#, Suning Chen^1, 2^#

**Affiliations:**

^1^ National Clinical Research Center for Hematologic Diseases, Jiangsu Institute of Hematology, First Affiliated Hospital of Soochow University, Suzhou, China

^2^ Institute of Blood and Marrow Transplantation, Collaborative Innovation Center of Hematology, Soochow University, Suzhou, China.

^3^ Yancheng No.1 People's Hospital, Affiliated Hospital of Medical School, Nanjing University, Yancheng Clinical College of Xuzhou Medical University, Yancheng, China

**Corresponding authors:**

Jia Yin

National Clinical Research Center for Hematologic Diseases, Jiangsu Institute of Hematology, First Affiliated Hospital of Soochow University, Institute of Blood and Marrow Transplantation, Collaborative Innovation Center of Hematology, Soochow University, Suzhou, China

Email: [yinjia@suda.edu.cn](mailto:yinjia@suda.edu.cn) Tel: +86 13771997357

Suning Chen

National Clinical Research Center for Hematologic Diseases, Jiangsu Institute of Hematology, First Affiliated Hospital of Soochow University, Institute of Blood and Marrow Transplantation, Collaborative Innovation Center of Hematology, Soochow University, Suzhou, China

Email: chensuning@suda.edu.cn Tel: +86 13814881746.

**
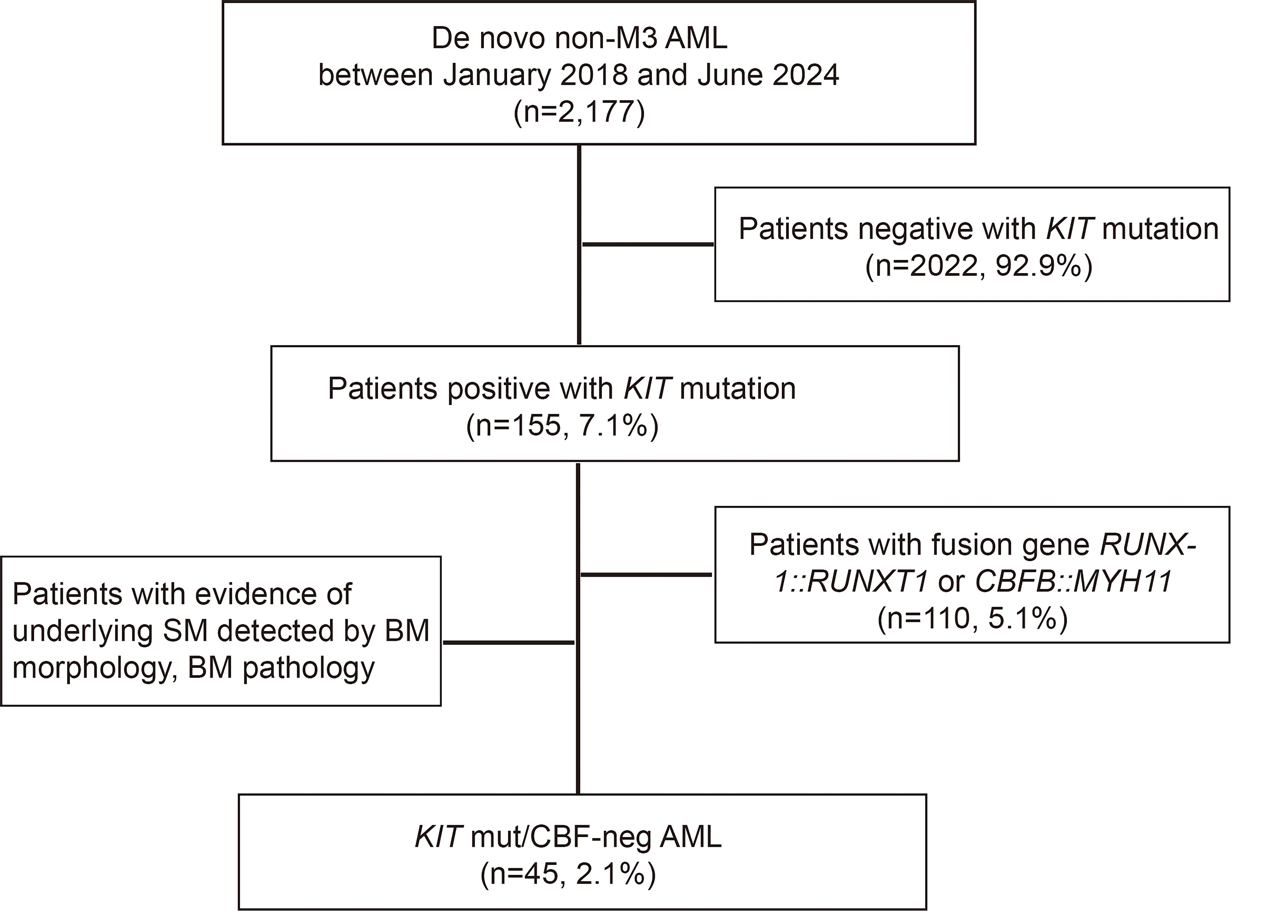
**

**Supplemental Figure 1** Flowchart of the study. AML: acute myeloid leukemia; BM: bone marrow; CBF: core binding factor; SM: systemic mastocytosis


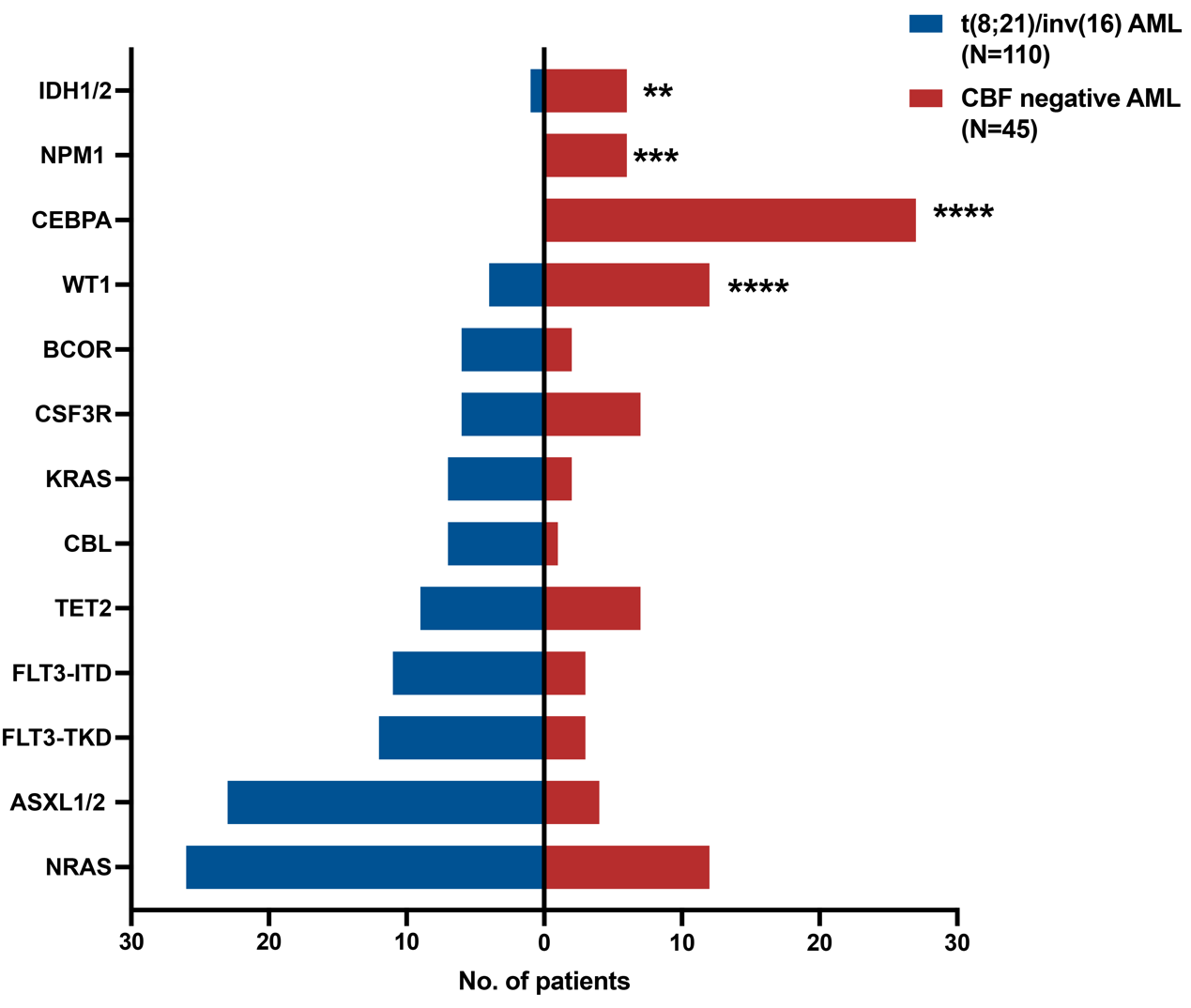


Supplemental Figure 2 Comparison of comutation between *KIT* mut CBF AML and *KIT* mut/CBF-neg AML. **, *p*<0.01; ***, *p*<0.001; ****, *p*<0.0001


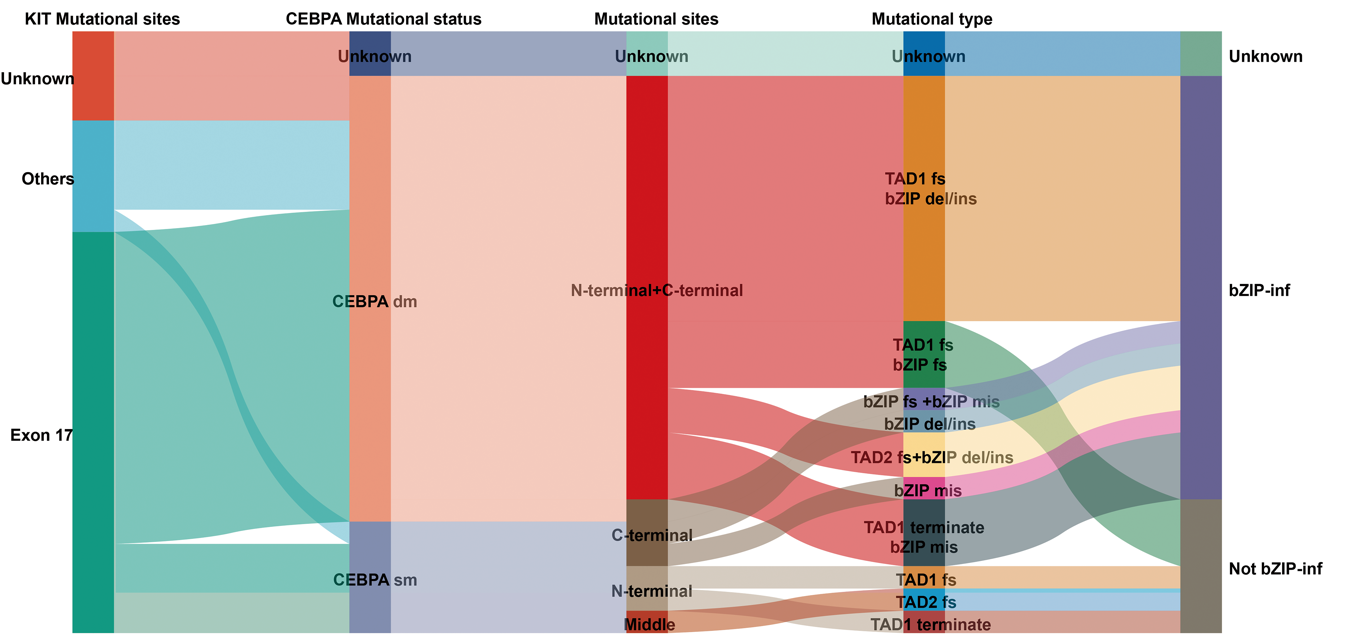


Supplemental Figure 3 Interrelationship of KIT mutational sites, CEBPA status, mutational sites and mutational type. bZIP-inf: bZIP in-frame; Fs: frameshift; del/ins: deletion or insertion; mis: missense

Supplemental Figure 4 Relapse-free survival (RFS) of the cohort (A) and patients with KIT exon-17 mutation and without (B).

Supplemental Figure 5 Event-free survival (EFS) and overall survival (OS) of patients with different *CEBPA* mutational status (A-D), NPM1 (E-F) and various ELN2022 risk stratification (G-H). Mut: mutated; WT: wildtype
