# Supplementary material for: KIT Mutant/Core binding factor-negative acute myeloid leukemia might be a complex subgroup with dismal prognosis: a single-center retrospective analysis
Source: Ann Hematol. 2026 Jan 20;105(2):42. doi: 10.1007/s00277-026-06814-7 (PMC12819538; doi:10.1007/s00277-026-06814-7)
Supplement: Supplementary file 2 — (DOCX 28.0 KB) [file 277_2026_6814_MOESM2_ESM.docx]

**Title:** *KIT* Mutant/Core Binding Factor-Negative Acute Myeloid Leukemia might be a distinct subgroup with dismal prognosis: a single-center retrospective analysis

**Journal:** Annals of Hematology

**Authors:**

Rui Jiang^1, 2#^, Zhibo Zhang^1, 2#^, Yizi Liu^1, 2#^, Wenqiang Qu^1, 2^, Zhao Zeng^1, 2^ , Linlin Wang^3^, Qian Wang^1, 2^, Jia Yin^1, 2^#, Suning Chen^1, 2^#

**Affiliations:**

^1^ National Clinical Research Center for Hematologic Diseases, Jiangsu Institute of Hematology, First Affiliated Hospital of Soochow University, Suzhou, China

^2^ Institute of Blood and Marrow Transplantation, Collaborative Innovation Center of Hematology, Soochow University, Suzhou, China.

^3^ Yancheng No.1 People's Hospital, Affiliated Hospital of Medical School, Nanjing University, Yancheng Clinical College of Xuzhou Medical University, Yancheng, China

**Corresponding authors:**

Jia Yin

National Clinical Research Center for Hematologic Diseases, Jiangsu Institute of Hematology, First Affiliated Hospital of Soochow University, Institute of Blood and Marrow Transplantation, Collaborative Innovation Center of Hematology, Soochow University, Suzhou, China

Email: [yinjia@suda.edu.cn](mailto:yinjia@suda.edu.cn) Tel: +86 13771997357

Suning Chen

National Clinical Research Center for Hematologic Diseases, Jiangsu Institute of Hematology, First Affiliated Hospital of Soochow University, Institute of Blood and Marrow Transplantation, Collaborative Innovation Center of Hematology, Soochow University, Suzhou, China

Email: chensuning@suda.edu.cn Tel: +86 13814881746.

Supplemental Table 1. Clinical characteristics between patients with non-intensive therapy (NIT) and intensive therapy (IST).

| Variables | NIT (n=12) | IST (n=31) | *p* |
| --- | --- | --- | --- |
| Male, n (%) | 6 (50) | 23 (74.2) | 0.16 |
| Age (years), median (range) | 54 (18-68) | 40 (17-66) | 0.12 |
| CBC at diagnosis, median (range) | |  |  |
| WBC , ×10^9^/L | 51.5 (3.2-239.5) | 6.7 (0.3-48.7) | 0.81 |
| Hb, g/L | 78 (54-103) | 93 (34-93) | 0.12 |
| PLT, ×10^9^/L | 60 (11-175) | 25 (3-1466) | 0.51 |
| LDH (U/L), median (range) | 664.9 (214.8-2363) | 521.9 (178-1925) | 0.55 |
| BM blast (%), median (range) | 79 (21-88) | 59 (13-92) | 0.10 |
| ELN 2022 risk stratification (n=43), n (%) | |  | 0.92 |
| Favorable | 6 (50.0) | 14 (45.2) |  |
| Intermediate/adverse | 6 (50.0) | 15 (48.4) |  |
| Gene comutation, n (%) |  |  |  |
| *CEBPA* bZIP-inf | 3 (25.0) | 13 (41.9) | 0.48 |
| *NPM1* | 4 (33.3) | 2 (6.5) | **0.04** |
| *FLT3*-ITD | 1 (8.3) | 2 (6.5) | 0.83 |
| *TP53* | 0 (0.0) | 1 (3.2) | 0.62 |
| Induction response, n (%) |  |  | 0.84 |
| CR or CRi | 10 (83.3) | 25 (80.7) |  |
| PR or NR | 2 (16.7) | 6 (19.4) |  |
| MRD negative after induction, n (%) | 10 (83.3) | 6 (19.4) | **0.0033** |
| Allo-HSCT | 8 (66.7) | 12 (38.7) | 0.17 |

**Abbreviations:** ANC: absolute neutrophil count; BM: bone marrow; bZIP-inf: bZIP in-frame mutations; CBC: complete blood count; Hb: hemoglobin; CR: complete remission; CRi: complete remission with incomplete hematologic recovery; LDH: lactate dehydrogenase; NR: no response; PLT: platelet; PR: partial remission; WBC: white blood cell count
